# Effects of Prenatal Phthalate Exposure on Thyroid Hormone Concentrations Beginning at The Embryonic Stage

**DOI:** 10.1038/s41598-017-13672-x

**Published:** 2017-10-12

**Authors:** Hui Gao, Wanke Wu, Yuanyuan Xu, Zhongxiu Jin, Huihui Bao, Peng Zhu, Puyu Su, Jie Sheng, Jiahu Hao, Fangbiao Tao

**Affiliations:** 1 Department of Maternal, Child & Adolescent Health, Anhui Medical University, Hefei, Anhui Province China; 2 Nanjing Maternity and Child Health Hospital, Nanjing, Jiangsu Province China; 3 Anhui Provincial Key Laboratory of Population Health & Aristogenics, Hefei, Anhui Province China

## Abstract

Limited studies have consistently shown an association of phthalates exposure with thyroid hormones (THs) in pregnant women. However, it remains unknown on which specific phthalates can affect THs and whether any effects could differ by gestational age. In the present study, we investigated associations between serum concentrations of phthalate monoesters [monoethyl phthalate (MEP), mono-(*n* + *iso)*-butyl phthalate (MBP) and mono(2-ethylhexyl) phthalate (MEHP)] and THs [thyroid-stimulating hormone (TSH), total thyroxine (TT4) and free thyroxine (FT4)] in Chinese pregnant women. 1,397 women were recruited from the China-Anhui Birth Cohort. Maternal serum samples were collected and used to measure phthalate metabolites and THs. Covariate-adjusted linear regression analyses showed that natural log (Ln)-transferred concentrations of MBP and LnMEHP were negatively associated with TT4 (*β* = −0.277 and –0.461, respectively; *p* < 0.001). Stratification analyses by gestational weeks showed significant associations of LnMBP and LnMEHP with TT4 in gestational weeks 5 to 8, 9 to 12, and 13 to 20. Our findings suggest an association of serum phthalates with lower TT4. The influence of MBP and MEHP on TT4 concentrations throughout the early pregnancy may begin from the embryonic stage (gestational weeks 5 to 8).

## Introduction

Phthalates are industrial chemicals that are extensively used as plasticizers in a variety of consumer products. For instance, diethyl phthalate (DEP) and di-n-butyl phthalate (DBP) are used for personal care and pharmaceutical products^1^. Di-(2-ethylhexyl) phthalate (DEHP) is usually used for polyvinyl chloride material, food packaging and medical device^2,3^. Because phthalates are not chemically bound to product structures, they release into environment such as air, water, and foods and become widespread contaminants. Human exposure to phthalates is ubiquitous^4–6^, and their metabolites have been detected in a wide range of biological samples including urine, blood, placenta, amniotic fluid, and breast milk among humans^7^.

Recently, attentions have been focused on the effects of various health risks in susceptible populations including pregnant women and infants^8,9^. An increasing number of studies have indicated that phthalates may disrupt the hormone system^10^. Limited studies^4,11–16^ have indicated that phthalates may have antagonistic effect on the thyroid function both *in vivo* and *in vitro* through a variety of mechanisms, such as down-regulation of the human sodium/iodide symporter (NIS) promoter^17^. It is well known that thyroid hormones (THs) deficiency during early pregnancy would result in neural deficits^18–22^. For instance, either low or high maternal free thyroxine (FT4) concentrations during early pregnancy (<18 weeks’ gestation) impairs IQ and decreases grey matter and cortex volume in children^19^. de Escobar *et al*. 2007^20^ reviewed that THs changes in mother during the first half of pregnancy potentially damaged fetal brain development. Therefore, exposure to phthalate may reduce fetal THs availability through maternal THs signaling^16,23,24^.

To date, a few studies have reported the potential association of phthalate exposure with thyroid function in pregnant women^13,16,25–28^. Huang *et al*. (2007; 2016)^13,28^ found urinary monobutyl phthalate (MBP) exposure was negatively associated with serum total thyroxine (TT4) and FT4 in early pregnancy. Johns *et al*. 2015^26^ observed an inverse association between DEHP and FT4 during gestational weeks 24 to 28. Similarly, our previous study^25^ showed negative associations of urinary monobenzyl phthalate (MBzP), mono(2-ethylhexyl) phthalate (MEHP) and mono(2-ethyl-5-hydroxylhexyl) phthalate (MEHHP) with serum TT4 and FT4, whereas positive associations of MEHP and MEHHP with thyroid stimulating hormone (TSH). However, Kuo *et al*. 2015^16^ observed positive correlations between MBP, MBzP, and mono(2-ethyl-5-oxohexyl) phthalate (MEOHP) and FT4 concentrations in the first trimester of gestation. A repeated measures analysis (median 10, 18, 26, and 35 weeks of gestation)^27^ revealed that MEHP and mono-3-carboxypropyl phthalate (MCPP) were positively related to TT4 and FT4, respectively, whereas MEHP, mono-iso-butyl phthalate (MiBP) and MCPP were negatively associated to TSH. Therefore, these findings suggested that the direction of the associations of THs phthalates with THs phthalates varied during pregnancy^27^. Which kind of phthalates and when phthalates affect THs are still inconclusive.

On the basis of the China-Anhui Birth Cohort (C-ABC), the objective of this pilot study was to investigate the cross-sectional association between prenatal serum concentrations of phthalate monoesters and maternal THs concentrations.

## Results

### Demographic characteristics

Among the 1,397 participants, the average age and gestational weeks of women were 26.6  ±  3.6 (range: 19–45) years and 12.0  ±  3.9 (range: 5−20) weeks, respectively. The pre-pregnancy body mass index (BMI) was 20.1  ±  2.3 kg/m^2^. Most women lived in urban areas (79.0%). Only 27.9% of women were none-passive smokers (Table 1). Pearson correlation and ANOVA tests showed that maternal age, pre-pregnancy BMI, gestational age, residence and passive smoking were associated with at least one type of THs.

**Table 1:** Demographic variables of the pregnant women and their associations with THs (*n*  =  1,397).

Characteristics	Mean ± SD or n (%)	Statistics (*p*-value)		
		LnFT4	TT4	LnTSH
Age (years)	26.6 ± 3.6	−0.002(0.945)	−0.077(0.004)	−0.071(0.008)
Pre-pregnancy BMI (kg/m^2^)	20.1 ± 2.3	−0.079(0.003)	−0.035(0.196)	0.016(0.541)
Gestational age (weeks)	12.0 ± 3.9			
5 to 8	263(18.8)	150.73(<0.001)	19.91(<0.001)	13.14(<0.001)
9 to 12	598(42.8)			
13 to 20	536(38.4)			
Residence				
Rural	294 (21.0)	3.62(0.057)	10.98(0.001)	0.019(0.889)
Urban	1103 (79.0)			
Education level (years)				
Low (≤9)	287(20.5)	1.20(0.301)	0.78(0.457)	1.14(0.321)
Middle (10–12)	385(27.6)			
High (≥13)	725(51.9)			
Family monthly income (RMB yuan)				
<1000	166(11.9)	0.48(0.620)	1.77(0.170)	0.64(0.527)
1000–1999	675(48.3)			
≥2000	556(39.8)			
Passive smoking				
Often	237(17.0)	0.13(0.876)	7.43(0.001)	0.39(0.678)
Sometimes	770(55.1)			
Never	390(27.9)			

Abbreviation: SD  =  standard deviation; BMI  =  body mass index.

Pearson correlation tests were used for maternal age and pre-pregnancy BMI; ANOVA tests were used for other categorical variables.

### Serum phthalate metabolites and THs concentrations

Phthalate metabolites and THs concentrations were measured in the serum samples (Table 2). All the three metabolites (MEP, MBP, and MEHP) were detectable in over 86% samples. The concentration of MEHP (median  =  6.14 ng/mL) was the highest, followed by that of MBP (median  =  5.97 ng/mL). The concentration of MEP (median  =  0.20 ng/mL) was the lowest. The median concentrations of FT4, TT4 and TSH were 0.96 ng/mL, 9.24 ng/mL, and 1.70 mIU/mL, respectively.

**Table 2:** Concentrations of phthalate metabolites and thyroid hormones in serum samples from the Chinese pregnant women (*n*  =  1,397).

Compounds	>LOD(%)	Percentiles						
		5^th^	10^th^	25^th^	50^th^	75^th^	90^th^	95^th^
Phthalate Metabolites								
MEP (ng/ml)	86	0.02	0.05	0.11	0.20	0.41	0.96	1.50
MBP (ng/ml)	100	1.11	1.69	2.91	5.97	11.39	19.85	26.53
MEHP (ng/ml)	100	1.60	2.09	3.27	6.14	11.23	17.28	20.97
Thyroid hormones								
FT4 (ng/dL)		0.73	0.77	0.86	0.96	1.07	1.18	1.28
TT4 (ng/dL)		6.63	7.21	8.24	9.24	10.68	12.06	12.75
TSH (mIU/L)		0.22	0.47	1.06	1.70	2.72	3.78	4.79

Abbreviations: LOD  =  limit of detection.

^*^ *p* < 0.05; ^**^ *p* < 0.001.

### Associations between serum phthalate metabolites and THs

Linear regression analyses (Table 3) showed that natural log (Ln)-transformed concentration of MBP (LnMBP) and LnMEHP were positively associated with LnFT4 (*β*  =  0.010, *p*  =  0.020 and *β*  =  0.012, *p*  =  0.031, respectively) after adjusting for potential confounders. LnMBP, LnMEHP, and LnPAE were negatively associated with TT4 (*β*  =  −0.277, −0.461, and −0.585, respectively; *p*  <  0.001).

**Table 3:** Linear regression analyses of associations between phthalate metabolites and THs.

	LnFT4		TT4			LnTSH	
	*β* (95%CI)	*p*	*β* (95%CI)		*p*	*β* (95%CI)	*p*
Crude models							
LnMEP	0.007(0.000,0.015)	0.067	−0.077(−0.157,0.004)		0.063	−0.019(−0.065,0.028)	0.432
LnMBP	0.015(0.006,0.025)	<0.001^**^	−0.301(−0.403,−0.199)		<0.001^**^	0.014 (−0.045,0.073)	0.642
LnMEHP	0.026(0.014,0.038)	<0.001^**^	−0.501(−0.624,−0.379)		<0.001^**^	−0.014(−0.085,0.058)	0.711
LnPAE	0.026(0.013,0.040)	<0.001^**^	−0.599(−0.738,−0.459)		<0.001^**^	0.004(−0.078,0.086)	0.929
Adjustment models							
LnMEP	0.006(0.000,0.013)	0.066	−0.067(−0.147,0.013)	0.099		−0.016(−0.062,0.030)	0.494
LnMBP	0.010(0.001,0.019)	0.020^*^	−0.277(−0.378,−0.176)	< 0.001^**^		0.019 (−0.040,0.078)	0.521
LnMEHP	0.012(0.001,0.023)	0.031^*^	−0.461(−0.585,−0.336)	< 0.001^**^		−0.000(−0.073,0.073)	0.999
LnPAE	0.011(0.000,0.023)	0.066	−0.585(−0.726,−0.445)	< 0.001^**^		0.030(−0.052,0.112)	0.468

Adjustment for maternal age, pre-pregnancy BMI, gestational weeks, residence and passive smoking.

^*^ *p*  <  0.05; ^**^ *p*  <  0.001.

### Stratification analyses based on gestational weeks

The addition of a product interaction term of gestational age with each phthalate metabolite to the model indicated that the associations of phthalate metabolites with TSH, FT4, or TT4 did not differ by time stages assessed in this study (data no shown). Stratification analyses (Table 4) were performed by gestational weeks. A positive association between LnMBP and LnFT4 (*β*  =  0.019, *p*  =  0.049), and an inverse association of LnMBP, LnMEHP, and LnPAE with TT4 (*β*  =  −0.211, −0.354, and −0.402, respectively; *p*  <  0.05) were observed as early as gestational weeks 5 to 8 and followed by gestational weeks 9 to 12 and 13 to 20. No significant association of any phthalate metabolites with TSH was observed.

**Table 4:** Stratification analyses of associations between phthalate metabolites and THs on basis of gestational weeks.

	LnFT4		TT4		LnTSH	
	*β* (95%CI)	*p*	*β* (95%CI)	*p*	*β* (95%CI)	*p*
**LnMEP**						
5−8 weeks’ gestation	0.012(−0.004,0.027)	0.145	0.097(−0.062,0.256)	0.231	−0.010(−0.104,0.085)	0.840
9−12 weeks’ gestation	0.008(−0.002,0.019)	0.129	−0.090(−0.215,0.034)	0.155	−0.052(−0.130,0.026)	0.192
13−20 weeks’ gestation	0.004(−0.008,0.015)	0.530	−0.088(−0.221,0.045)	0.194	−0.011(−0.079,0.057)	0.746
**LnMBP**						
5−8 weeks’ gestation	0.019(0.000,0.038)	0.049^*^	−0.211(−0.403,−0.020)	0.031^*^	0.100(−0.013,0.214)	0.083
9−12 weeks’ gestation	0.015(0.002,0.029)	0.029^*^	−0.272(−0.431,−0.114)	0.001^**^	−0.029(−0.129,0.071)	0.568
13−20 weeks’ gestation	0.001(−0.014,0.015)	0.994	−0.277(−0.450,−0.105)	0.002^*^	0.017(−0.072,0.105)	0.710
**LnMEHP**						
5−8 weeks’ gestation	0.021(−0.002,0.044)	0.069	−0.354(−0.581,−0.126)	0.002^*^	0.014(−0.123,0.152)	0.837
9−12 weeks’ gestation	0.009(−0.008,0.026)	0.291	−0.521(−0.713,−0.329)	<0.001^**^	−0.002(−0.125,0.120)	0.968
13−20 weeks’ gestation	0.009(−0.009,0.027)	0.325	−0.454(−0.668,−0.241)	<0.001^**^	−0.014(−0.124,0.097)	0.807
**LnPAE**						
5–8 weeks’ gestation	0.024(−0.002,0.050)	0.071	−0.402(−0.661,−0.143)	0.003^*^	0.119(−0.037,0.275)	0.133
9−12 weeks’ gestation	0.019(0.000,0.038)	0.052	−0.588(−0.808,−0.369)	<0.001^**^	−0.040(−0.180,0.100)	0.573
13−20 weeks’ gestation	−0.003(−0.023,0.018)	0.804	−0.578(−0.820,−0.337)	<0.001^**^	0.010(−0.116,0.135)	0.879

Adjustment for maternal age, pre-pregnancy BMI, residence and passive smoking.

^*^ *p*  <  0.05, ^**^ *p*  <  0.001.

## Discussion

In the present study, the detection rates of the three phthalate metabolites in serum were 86%−100%. The medians of serum MEP, MBP, and MEHP were 0.2, 5.97, and 6.14 ng/mL, respectively. The result suggested that Chinese women during gestational weeks 5 to 20 were widely exposed to low concentration of DEP, DBP, and DEHP. It was expected that concentrations of phthalate metabolites in urine were higher than those in serum. However, our reported concentrations of serum phthalates were different from those in other studies. The geometric means of serum MEP, MBP, and MEHP were 67.8, 14.7, and 1.39 ng/mL respectively, in females from the 2009–2010 National Health and Nutrition Examination Survey^29^. An infant congenital hypothyroidism study in Korea^30^ observed that the mean plasma concentrations of MBP and MEHP were 19.87 and 9.82 ng/mL in mothers from control group, and 27.38 and 13.57 ng/mL in mothers from case group, respectively. The variation of phthalate exposure levels was partially attributed to the variations in sample collection time (e.g., gestational weeks)^31^ and the differences in laws and regulations against application of phthalates in different countries.

Our results showed negative associations of maternal serum LnMBP and LnMEHP with TT4 concentrations, which occurred as early as gestational weeks 5 to 8. Similarly, the association of “LnPAE” with TT4, which combined the three metabolites into a single class of chemicals, was also identified. Positive associations of serum LnMBP and LnMEHP with LnFT4 were also found. Data on the association of serum phthalates with thyroid function in pregnant women are limited. Previous animal studies showed that rats fed DEHP through the diet displayed histopathologic alterations in the thyroid indicative of hyperactivity, increased thyroglobulin turn over, and significantly decreased plasma T_4_ concentrations^13,32–34^. A few epidemiological studies explored the associations of prenatal phthalates exposure with maternal thyroid function, which was assessed by metabolites concentrations in urine samples^13,16,25–28^. Three studies reported that maternal phthalates exposures (e.g., MBP, MBzP, MEHP and MEHHP) were associated with reduced concentrations of TT4^13,25,28^, which were consistent with our results. However, Johns *et al*. 2016^27^ reported that increased MEHP was associated with an increase of TT4. No significant associations of maternal TT4 concentrations were found by Kuo *et al*. 2015^16^ and John *et al*. 2015^26^. Three previous studies revealed that some phthalates (e.g., MBP, MBzP and metabolites of DEHP) exposure were related to decreased FT4 concentrations^13,25,26^, whereas two investigations reported opposite results^16,27^. No significant relationships were observed for TSH in most previous studies. Our previous study^25^ found that MEHP and MEHHP were positively associated with TSH, while Johns *et al*. 2016^27^ observed an inverse association of MEHP, MiBP, and MCPP with TSH. Obviously diverse relationships between phthalates and THs were showed. We speculated that there were several potential reasons for these inconsistent findings. First, these studies were based on different sample sizes. Only our previous study recruited over 500 women (n  =  2,521)^25^. Second, urine samples were collected in different time/gestational weeks. Two studies^16,25^ obtained urine samples in the first trimester of pregnancy, and three studies^13,26,28^ in the second trimester of pregnancy. Johns *et al*. 2016^27^ averagely collected urine in 10th, 18th, 26th and 35th weeks’ of gestation for a repeated measures analysis.

The biological mechanisms of above abservations still remain unclear. Several studies have shown that thyroid hormone receptors (TRs) may be unintended targets of manufactured chemicals. Phthalates also have been reported to have TRs antagonist activity^11–16^ and/or interfere triiodothyronine binding to transthyretin^35^. Some phthalates such as DBP and DEHP may induce thyroid hyperactivity and decrease thyroxin concentrations through modulating the transcriptional activity of NIS^36^.

There are some limitations in the present study. Firstly, we did not collect urine samples. Urine is the best matrix for measuring non-persistent chemicals including phthalates^37^. However, phthalate metabolites concentrations in serum significantly correlated to those in urine, suggesting that metabolites in serum could be used as biomarkers of human exposure^38^. Secondly, as the present study is a pilot research, the indicators of thyroid function and phthalates exposure were limited. We did not measure thyroid peroxydase antibody which was one of the most important determinants of thyroid function. In addition, only the primary metabolites of DEP, DBP and DEHP were analyzed since pregnant women were predominantly exposed to these three types of phthalates^13^. Finally, the cumulative exposure risks of multiple other phthalates could not be obtained in the present study.

Our findings indicate that serum LnMBP and LnMEHP during early pregnancy are associated with decreased maternal TT4 concentrations. Besides, it is suggested that the effects of MBP and MEHP exposure on TT4 could begin as early as at the embryonic stage.

## Materials and Methods

### Study participants

The C-ABC study was conducted to examine the delayed, cumulative and interactive effects of maternal environmental exposures on birth outcomes and children’s development. All details of in- and exclusion criteria were described in our previous studies^39,40^. Briefly, this cross-sectional survey, as a part of the C-ABC study, enrolled participants in the first 20 weeks’ gestation in Ma’anshan city between November 2008 and September 2009. 1,599 women with singleton pregnancies were invited to participate at their first antenatal care visit. The response rate was 97.4% (*n*  =  1,557). They were required to complete a self-administered questionnaire. Meanwhile, a single blood sample was collected from each woman for the laboratory examination. Finally, 1,397 pregnant women with complete data of both THs (measured in 2010) and phthalates (measured in 2012) were enrolled in the present study.

Informed consent was obtained from all participants in our study. The study (including the additional serum analysis of phthalates and thyroid hormones) was approved by the Biomedicine Ethical Committee of Anhui Medical University (approval NO. 2007002). All the study methods were performed in accordance with Helsinki declaration II.

### Covariates

Possible confounding variables of basic demographic characteristics were obtained, including maternal age (continuous), pre-pregnancy body mass index (BMI; continuous), gestational age (continuous), residence (rural or urban), educational level (below ≤9 years, middle 10–12 years or high ≥13 years), family monthly income (<1000, 1000–1999 or ≥2000), and passive smoking (often, sometimes, or never). The multiple imputation method was used to impute missing values.

### Serum phthalate monoester analyses

At the prenatal visit, a single peripheral venous blood sample per woman was drawn from an antecubital vein into 10 mL glass vacuum vessels. After centrifugation, samples were aliquoted and stored at −80 °C until biochemical analyses. In the Anhui Provincial Key Laboratory of Population Health & Aristogenics (Anhui Medical University, Hefei City, China), a revised isotope dilution-solid phase extraction (SPE) coupled to high-performance liquid chromatograph-tandem mass spectrometry (HPLC-MS/MS; HPLC, Agilent 1200; MS, Agilent 6410) method (Frederiksen *et al*., 2010) was used to analysis the serum concentration of phthalates. In brief, monoethyl phthalate (MEP) was measured through external standard method, while MBP (mono-*n*-butyl phthalate plus mono-iso-butyl phthalate) and MEHP were measured through isotope-labeled internal standard (MBP-d_4_ and^13^C-MEHP; Cambridge, MA, USA) method.

### Serum Thyroid hormones analyses

Concentrations of TT4, FT4 and TSH in maternal serum samples were assayed in the Department of Clinical Laboratory in the First Affiliated Hospital of Anhui Medical University in October 2010, using chemiluminescent immunoassays on an automated platform (LIAISON analyzer; DiaSorin SpA., Saluggia, Italy). The functional sensitivity of TSH was 0.004 mIU/L. The detectable concentrations of TSH, free T_4_, and total T_4_ were 0.004–100 mIU/L, 0.1–10 ng/dL and 3.0–20 ng/dL, respectively, as described in the previous study^40^.

### Statistical analyses

All statistical analyses were performed using the Statistical Package of Social Sciences (SPSS, version 13.0; SPSS, Inc., Chicago, IL).

The phthalate monoester concentrations below the LOD were replaced with a value of LOD/√2 for statistical analyses. To investigate the combined risks of multiple phthalate exposure, we calculated a single variable “PAE” through summing up the concentrations of all metabolites by weighting their molecular weight. Here is the equation.

$$PAE=\frac{MEP}{M{W}_{MEP}}+\frac{MBP}{M{W}_{MBP}}+\frac{MEHP}{M{W}_{MEHP}}$$

Firstly, we statistically described the concentrations of phthalate metabolites using several selected percentiles. Furthermore, as the distributions of phthalate metabolites and FT4 and TSH were right-skewed, their concentrations were natural logarithm (Ln)-transformed for statistical analyses. Multivariable linear regression analyses were used to explore the relationships between phthalate monoesters and THs. Finally, stratification analyses were applied by gestational weeks of serum sampling time (gestational weeks 5 to 8, 9 to 12 and 13 to 20). Potential confounders which were significantly correlated to THs according to Pearson correlation or ANOVA tests were included in the adjusted statistical models.
